# The Book World of Medicine and Science

**Published:** 1893-10-14

**Authors:** 


### THE BOOK WORLD OF MEDICINE AND SCIENCE.

AN ELEMENTARY TEXT BOOK OF BIOLOGY, COMPRISING VEGETABLE AND ANIMAL MORPHOLOGY AND PHYSIOLOGY. By J. R. AINSWORTH DAVIS, B.A., Trinity College, Cambridge, Professor of Biology and Geology in the University College of Wales, Aberystwyth. (London: Charles Griffin and Co., Limited, Strand.)

Specialisation is the curse as well as the strength of modern methods of advance, whether in science, literature, or art; but it is perhaps in science that this method has been driven almost to an illogical limit. What a satire on the ingenuous enterprise of John Hunter, who sought to found a museum "for everything scientific," is the special department of our modern museum. Each special department is divided and sub-divided, and each sub-division has its own special custodian; but the custodian of Block A, be he never so cunning a "savant" of his own special department, rises altogether superior to an intelligent interest in the affairs of Department B, although the two collections may be closely and intimately associated. And as it is with our modern museums so it is with modern teachers of science. In these days of one man one subject, we wait in vain for some Hunter, some Linnæus, or even a second Darwin to rise a giant among us, who with the master hand of a constructive builder shall synthesise and weld together into a comprehensive and intelligible whole the scattered but priceless fragments of knowledge carved out by individual workers in the thousand workshops of science. Perhaps our brains are not large enough to appreciate the strides of science, much less to keep pace with the rate of modern scientific research. Very refreshing, therefore, is it to recognise even a humble attempt at a reversion to the old system before specialisation became the necessity of the hour, and to contemplate the ambitious expanse of title in this little work before us. It boldly styles itself a text book of vegetable and animal morphology and physiology, and in six hundred pages it attempts to wrestle with the difficulties of the questions involved in what may be called, according to modern lights, four distinct subjects. It has, however, attained to the dignified position of a second edition, and has doubtless helped many a student to meet the exacting requirements of medical and—we draw the distinction—Scientific Examining Board, and hence we do not desire to pour cold water on what is really a very praiseworthy attempt at consolidating, for the benefit of beginners, the scattered information on these subjects. The method of the book is concise, business-like, and clear, and the schematic handling of such complicated subjects as the comparative development in the male and female of the genito-urinary system, and the successive stages in the life history of the liver fluke, are exceedingly well done; but where the book fails, and that hopelessly, and shows the want of philosophic as well as literary handling, is in those paragraphs which treat of the really important and fundamental biological problems such as the nature of protoplasm, the process of nuclear and cellular division, and the part that chlorophyll plays in the economy of vegetable life. They are, of course, the natural stumbling blocks of all who try to treat of these subjects; but whether it be teacher or student who examines the pages of this work in the hope of finding clearer views and new lights on these difficult questions, he must turn aside

THE BOOK WORLD OF MEDICINE AND SCIENCE—*continued.*

with a sigh of disappointment and regret that one more unhappy author has been weighed in the scales and found wanting.

This, the second edition, has been revised and enlarged, and a chapter on the distribution of animals interpolated. This section, if of no artistic merit, is at least accurate and reliable, and we predict for this work a permanent and honoured position on the shelves of every scientific library.

Lectures on Sanitary Law. By A. Wynter Blyth, M.R.C.S. (England); L.S.A. (London); Fellow of the Institute of Chemistry; Barrister-at-Law, of Lincoln's Inn; Professor of Hygiene, College of State Medicine. (London: Macmillan and Co.)

Very few people know the legal aspects of the health question, their rights and their wrongs, their duty to their neighbour, and their neighbour's duty to them. Many excellent laws remain almost as dead letters, because the people who are most concerned in profiting by them do not know of their existence, or at least have no notion how to set them in action. Sanitary laws are most apt to be ignored in the midst of an ignorant population, and even those who complain of, and suffer from their neighbour's habit of—say, rearing pigs in the back garden, or choking drains with all manner of refuse, which sewers were never meant to carry away, do not know how to get their wrongs set right. The inspector of nuisances may keep as keen a look-out as possible, but the inspector of nuisances must in the majority of cases, be led by his nose, and non-odorous dangers escape without suspicion. In the matter of notifying infectious disease how much mischief may be done before the proper authorities learn of the evil! What toleration of noxious trades may be granted by those who do not know they can be suppressed! What faults in building and lack of sanitary conveniences may be endured when the occupants of a house are ignorant of what a builder or proprietor is compelled by law to do!

Mr. Wynter Blyth's lectures put together in a clear and comprehensible form, a great amount of information which has hitherto been shut up in statutes and law text-books. The cases given, and the decisions of judges quoted, give an effective interpretation of the law when its real nature might be a little obscure to those who have not to deal with it frequently. To medical officers of health, this volume should be invaluable, indeed it may be regarded as indispensable; and to all who are interested, officially or otherwise, in the health and welfare of the community, it must prove of the highest utility. In its own sphere it will certainly command a permanent place, but we could wish that the sphere were likely to be larger. Until every householder is a sanitarian, the hygienic millennium can hardly be hoped to come. Would it not be possible for Mr. Wynter Blyth to compile a smaller edition of his lectures, including only the things most necessary to domestic sanitation, and the laws relating to it, and to publish a handy volume, a shilling manual, or something of the sort, which would have a fair chance of finding its way into every home? We believe that such a book would be immensely useful to the public; we do not think it would be unprofitable to either author or publisher.

OCTOBER REVIEWS.

The National Review is full of interesting matter this month. Mr. Leslie Stephen's well-balanced and judicial essay on the art of biography will attract many readers. Mr. Alfred Austen's charming vision of "The Garden that I Love,"

THE BOOK WORLD OF MEDICINE AND SCIENCE—(*continued*).

gives the essence of this unique and never to be over-praised summer. Other reminiscences of the holidays are Mr. Rees's "Fortnight in Finland" and the Hon. Alfred Lyttleton's attempt to explain the fascinations of golf.

In the New Review two medical articles attract attention. An eminently inconclusive inquiry by Mr. Dunn into "The Increase of Cancer," and a revival by Mr. Adolphe Smith of the old but not yet settled question, "Are We Prepared to Resist a Cholera Epidemic?" Mr. Smith thinks the public is too easily content with protecting the drinking water alone, and calls attention to the fact that in studying the cholera outbreak at Havre on the spot he found the water supply above suspicion. The real source of danger, he contends, is the drainage, owing to the fact that the "comma bacillus" is found in infected districts to be abundantly present in persons affected only by slight attacks of diarrhœa, or even in perfect health. In support of this, he quotes the following incident. "During the cholera epidemic some medical men came to Toulon from Montpellier to help to nurse the sick. At Montpellier there was no cholera. After these doctors had remained some time at Toulon, and though they were still enjoying good health, the idea occurred to them to examine their own dejections. They were not a little surprised and alarmed to find any number of comma bacilli! Thus if these experiments were correctly made, not only may a person with a slight attack of diarrhœa become the point of departure for a cholera epidemic, but a person in apparently perfect health may spread the fell disease. Some one from Grimsby, in perfect health, or perhaps affected by some very slight intestinal disturbance, may have introduced the specific germs of cholera into the drains of the inn at Ashbourne. These drains leaked and contaminated the well in the yard of the inn. Fifteen persons drinking the water suffered from cholera, and nine died." After all, then, the purification of the water supply remains the great precept of "Inspector Cholera."

There is nothing very striking in the Nineteenth Century. Professor Mavor has a somewhat depressing inquiry into the measure of success likely to be attained by the new schemes for "Setting the Poor on Work" by inducing them to return to the land. He fails to discriminate between the creation of a pauper class of labourers which the old system aimed at, and the awakening of a spirit of independence and self-reliance which recent experiments have shown to be possible, even among the most degraded, when placed in new surroundings.

Mr. Arnold White has a very strong article in the Fortnightly on this same subject of "The Unemployed," directed fiercely, yet not altogether unsympathetically, against the indiscriminate almsgiving which fosters the evil.

Every member of the profession will of course read Sir William Dalby's "Letters on Medicine as a Career," of which the first instalment appears in Longman's Magazine this month. The implied comparison to Dr. Chesterfield is perhaps a little unfortunate, but every line is full of interest, and the allusions to living celebrities will be keenly relished by the initiated.

